# Oligomeric tau-targeted immunotherapy in Tg4510 mice

**DOI:** 10.1186/s13195-017-0274-6

**Published:** 2017-06-27

**Authors:** Sulana Schroeder, Aurelie Joly-Amado, Ahlam Soliman, Urmi Sengupta, Rakiz Kayed, Marcia N. Gordon, David Morgan

**Affiliations:** 1 Byrd Alzheimer’s Institute and Department of Molecular Pharmacology and Physiology, University of South Florida, 4001 E. Fletcher Ave, Tampa, FL 33613 USA; 2 George P. and Cynthia Woods Mitchell Center for Neurodegenerative Diseases, Departments of Neurology, and Neuroscience and Cell Biology, University of Texas Medical Branch, Galveston, TX 77555 USA; 3 Department of Psychiatry and Behavioral Neuroscience, University of South Florida, Tampa, FL 33612 USA

**Keywords:** Tau, Immunotherapy, Tg4510 mouse, Oligomers

## Abstract

**Background:**

Finding ways to reverse or prevent the consequences of pathogenic tau in the brain is of considerable importance for treatment of Alzheimer’s disease and other tauopathies. Immunotherapy against tau has shown promise in several mouse models. In particular, an antibody with selectivity for oligomeric forms of tau, tau oligomer monoclonal antibody (TOMA), has shown rescue of the behavioral phenotype in several murine models of tau deposition.

**Methods:**

In this study, we examined the capacity of TOMA to rescue the behavioral, histological, and neurochemical consequences of tau deposition in the aggressive Tg4510 model. We treated mice biweekly with 60 μg TOMA i.p. from 3.5 to 8 months of age.

**Results:**

Near the end of the treatment, we found that oligomeric tau was elevated in both the CSF and in plasma. Further, we could detect mouse IgG in Tg4510 mouse brain after TOMA treatment, but not after injection with mouse IgG1 as control. However, we did not find significant reductions in behavioral deficits or tau deposits by either histological or biochemical measurements.

**Conclusions:**

These data suggest that there is some exposure of the Tg4510 mouse brain to TOMA, but it was inadequate to affect the phenotype in these mice at the doses used. These data are consistent with other observations that the rapidly depositing Tg4510 mouse is a challenging model in which to demonstrate efficacy of tau-lowering treatments compared to some other preclinical models of tau deposition/overexpression.

## Background

Tau is a microtubule binding protein, which assists in maintaining the physical structure of neurons, primarily the axons. Tau also facilitates trafficking of organelles and intracellular compounds within the cell in its normal state [1]. This is an important protein for normal cell functioning, but can become pathological. This pathology is associated with various post-translational modifications, most notably hyperphosphorylation. As tau becomes hyperphosphorylated, it misfolds and aggregates into oligomers, and ultimately fibrils. These aggregated forms of tau are associated with a class of neurodegenerative disorders called tauopathies, which include fronto-temporal lobe dementia, Pick’s disease, corticobasal degeneration, argyrophilic grain disease, and Alzheimer’s disease (AD). These diseases have different origins and symptoms, but all have accumulation of aggregated forms of tau as a common feature.

Tau is an attractive target to treat because the progressive pathology of the protein highly correlates with AD symptoms [2, 3]. Amyloid beta (Aβ) was initially investigated, due to the linkage of amyloid metabolism to genetic forms of AD [4]. One of the first approaches to reducing brain Aβ was the use of immunotherapy [5, 6] and this approach has advanced to phase 3 clinical testing [7, 8]. The success of immunotherapy in preclinical models of amyloid deposition also led to later attempts to pursue immunotherapeutic approaches to tau deposition (reviewed in [9]).

Traditionally, tau has been considered a presynaptic protein because it stabilizes microtubules and assists in transport through the axon. However, there have been recent studies that have identified mechanisms for tau to be transmitted across synapses to nearby post-synaptic cells, which serves as a means for understanding progressive tau pathology and spread [10, 11]. Dendritic tau has been associated with synaptic disruption [12, 13].

Immunotherapy has become a focus to attempt to treat tauopathies and other protein-based neural diseases [14], especially given the successes in using this strategy for treating the Aβ component of AD [15]. Immunotherapy can either be “active,” where an antigen is delivered to the body which then produces its own antibodies to effect healing, or “passive,” in which actual antibodies are administered like a drug. The former is more likely to produce side effects, whereas the latter requires more frequent administration to maintain effective titers of the treatment antibody. Both strategies have been investigated extensively in the past several years.

Our laboratory has examined several antibodies directed against tau (focusing on passive immunization), in an effort to find the most successful candidate to ameliorate pathology and behavioral deficits in Tg4510 mice. In this study, we utilized passive immunization of a tau oligomeric monoclonal antibody (TOMA), which was provided by Rakez Kayed’s laboratory [16, 17]. This antibody had shown positive effects in JNPL3 mice and hTau mice, but had not been tested in Tg4510 mice. JNPL3 mice slowly develop tau aggregation and deposition in the spinal cord and brainstem with small amounts of forebrain pathology. The Tg4510 mice rapidly accumulate tau deposits in forebrain regions overlapping CaM kinase II distribution. There is also considerable atrophy and neuron loss in the Tg4510 forebrain with aging, while there is less such pathology in the JNPL3 model. hTau mice develop pathology even later than the JNPL3 mice [18].

## Methods

### Animals

The Tg4510 mouse line (0N4R P301L) was chosen for these studies because these mice are known to have tau pathology (expression of the P301L mutation) directed to the forebrain, which more closely resembles an AD type of tauopathy [19]. Of note, the mutant human tau expression can be suppressed by application of doxycycline. Tg4510 mice, and their littermates, were bred locally by crossing mice transgenic for a tetracycline-operon-responsive element (tTA), controlled by a calmodulin kinase II (CAMK II) promoter, with a separate mouse line transgenic for human P301L tau driven by the Tet operon, as described previously [19]. The resulting cross yields approximately 25% nontransgenic (nTg) mice, 25% P301L-only (tau-only) mice, 25% tTA-only (Tet) mice, and 25% Tg4510 (tau-tet) mice. For this study, while the Tg4510 mice were the primary interest, we also used nontransgenic (nTg) and Tet-only littermates as controls. The mice were approximately 3.5 months old at first injection and 8 months old at the end of treatment. Groups were as follows: nTg + saline, *n* = 10; Tet + saline, *n* = 10; Tg4510 + IgG1, *n* = 9; and Tg4510 + TOMA, *n* = 9.

### Antibody treatment

In this study, we focused on one antibody, TOMA [17]. We examined the effects of systemic administration to Tg4510 mice, to evaluate whether the antibody could prevent behavioral and pathological components of the Tg44510 phenotype. Mice were injected i.p. with 60 μg TOMA or IgG control, every 2 weeks for a total of 10 injections. Tet-only and nTg littermates were also injected at these times with saline to control for effects of biweekly injections on behavioral measures. The experimenter performing the injections was blind with respect to treatment condition. Body weight and food intake were measured at each injection time, to identify whether the treatments were altering either of these metabolic measures. We noted that the Tg4510 mice consumed significantly more food, but also exhibited a significant reduction in body weight likely due to their excessive activity as described previously [20, 21]. However, neither body weight nor food intake was modified by the antibody treatment.

### Behavioral testing

Animals were tested at 7.5 months for general activity levels by open-field testing, followed by rotarod and Y-maze tests. Y-maze testing indicates overall activity, or hyperactivity, based on the number of arm entries. It can also give a general indication of working memory when one analyzes the arm alternations. Mice were also tested for cognitive function using novel object recognition, spatial navigation memory using the 2-day radial arm water maze [22] followed by 1 day of reversal training [23], and associative learning using fear conditioning. Mice were also tested for novel mouse recognition (described in [20]), using a modified version of a social interaction task. Open field and novel object recognition results were quantified using Anymaze software (Stoelting Co., USA).

### Tissue collection and staining

One day following the last antibody injection, the animals were euthanized using Somnasol (pentobarbital, phenytoin, EtOH, propylene glycol, rhodamine, and benzyl alcohol). Anesthetized mice had CSF removed by penetrating the cisterna magna with a glass capillary tube (Sutter Instruments, USA) following the method described previously [24]. Blood was collected by intracardiac puncture using EDTA and then centrifuged at 1000 × *g* for 15 min at 4 °C for plasma collection. Mice were then perfused with buffered normal saline on a heated pad to prevent reductions in body temperature and artifactual tau phosphorylation. The brain was exposed and the right hemisphere was collected, preserved in freshly prepared 4% paraformaldehyde in Dulbecco’s phosphate-buffered saline. Twenty-four hours later the brains were cryoprotected by successive incubations of 10%, 20%, and 30% sucrose solutions for 24 h each. The left hemisphere was dissected into the following regions: anterior cortex, posterior cortex, hippocampus, striatum, thalamus, cerebellum, and rest of brain. All dissected tissue samples were snap-frozen on dry ice, and then stored at −80 °C until ready for use.

Horizontal sections (25 μm thick) of the left hemisphere were collected into 24-well plates. For each staining performed, eight sections spaced roughly 600 μm apart were used. Gallyas silver staining for neurofibrillary tangles was conducted as described previously [20]. Free-floating immunohistochemistry was completed for H150 (Santacruz Biotechnology, Dallas, TX, USA), tau phosphorylated at Ser199/202 (pSer199/202; Anaspec, Fremont, CA, USA), pSer396 (Anaspec), and the microglial marker CD45 (Invitrogen, Carlsbad, CA, USA) [25]. Additionally, we examined the staining for mouse IgG with biotinylated horse anti-mouse IgG specific secondary antibody (Vector Laboratories, Burlingame, CA, USA) to detect whether the systemically injected antibodies may have bound to brain tissue.

Stained anatomical sections were scanned digitally using a Zeiss slide scanner followed by quantification of stained area in user-defined regions of interest with custom-designed software. Values for all sections from the same mouse were averaged to represent a single value for that region in subsequent statistical analysis.

For immunofluorescence, frozen cryostat sections from 5-month-old Tg4510 mice were fixed in chilled methanol and then blocked with goat serum for 1 h. Sections were incubated with TOMA (1:50) overnight. The following day, the sections were washed three times with PBS, goat anti-mouse IgG Alexa-568 (1:700; Invitrogen) was added, and the sections were incubated for 1 h at room temperature. The sections were washed three times with PBS and incubated overnight with Tau5 (1:300) at 4 °C. The sections were then washed three times followed by incubation with goat anti-mouse IgG Alexa-488 for 1 h at room temperature. Sections were then washed, stained with DAPI (Vector Laboratories), and mounted in Vectashield mounting medium (Fluoromount-4′,6-diamidino-2-phenylindole). Stained slides were imaged with an epifluorescence microscope (Nikon Eclipse 800) equipped with a CoolSnap-FX monochrome CCD camera (Photometrics) using standard Nikon FITC and DAPI filters.

### Biochemistry/western blotting

Dissected brain regions were homogenized and then sonicated in RIPA buffer, containing protease inhibitor cocktail (Sigma Aldrich) and phosphatase inhibitor cocktails I and II (Sigma Aldrich) as per the manufacturer, and centrifuged at 40,000 × *g* for 30 min at 4 °C. The supernatant was collected and protein concentrations were determined by the BCA protein assay kit (Pierce, Rockford, IL, USA). The resulting pellet was digested with 70% formic acid according to the wet tissue weight, and then neutralized with NaOH to analyze RIPA-insoluble proteins. Equal amounts of proteins (5 μg/well for soluble fraction, 2 μg/well for insoluble fraction) were loaded in each well of 4–12% Bis–Tris gels, transferred to a 0.2-μm pore size nitrocellulose membrane and immunoblotted with H150 (Santacruz Biotechnology), pSer199/202 (Anaspec), and pSer396 and pser262 (Anaspec) at 1:1000-fold dilution. Fluorescently tagged secondary antibodies (LI-COR Biosciences) were used at a dilution of 1:10,000. Western blot results were quantified by scanning with a LI-COR Odyssey fluorescent scanner. AlphaEase FC software was used to normalize the tau bands to actin for the soluble fraction. Normalization of the insoluble fraction was conducted using REVERT reagent (LI-COR) to measure total protein. Statistical analysis was then conducted using StatView.

A slightly different protocol was used for staining with the TOMA antibody. Forty micrograms of the PBS soluble fractions from Tg4510 brain homogenates were loaded into the wells of precast NuPAGE 4–12% Bis–Tris gels (Invitrogen) for electrophoresis. After transferring the proteins onto nitrocellulose they were blocked with 10% nonfat dry milk overnight at 4 °C. The membrane was incubated with TOMA antibody diluted in 5% milk solution (1:100) for 1 h at room temperature. TOMA immunoreactivity was detected with an HRP-conjugated anti-mouse IgG secondary antibody by incubating for 1 h at room temperature (1:3000; Southern Biotech). The bands were visualized with ECL plus (GE Healthcare).

### ELISA for tau oligomers

ELISA plates (Nunc immobilizer, 96 wells, amino modules; Thermo Fisher Scientific) were coated with 0.05 M sodium bicarbonate (pH 9.6) as a coating buffer and 2 μl of either CSF or plasma sample was added in duplicate. The plates were incubated overnight at 4 °C and then washed once with 1× TBS containing 0.01% Tween 20. Plates were blocked with 10% nonfat dry milk solution at room temperature for 2 h followed by incubating with T22 antibody diluted 1:250 in 5% nonfat dry milk solution for 1 h at room temperature. HRP-conjugated anti-rabbit IgG secondary antibody (Southern Biotech) was used to detect the T22 immunoreactivity in 1:3000 dilution. After washing, the plates were incubated with 3,3,5,5-tetramethylbenzidine (TMB-1 component substrate; DAKO) in the dark and the reaction was finally stopped by adding 2 M HCl solution. Colorometric reaction was analyzed with a Polar Star Omega plate reader (BMG Labtech) at 450 nm wavelength.

### Statistical analysis

In general, results were analyzed by one-way ANOVA with subsequent means comparisons made using Fisher’s test for multiple comparisons. In some cases where tau was measured, the absence of any signal in nTg mice led to using *t* tests in Tg4510 groups only.

## Results

### Detection of antibody in the brain

**Fig. 1:**
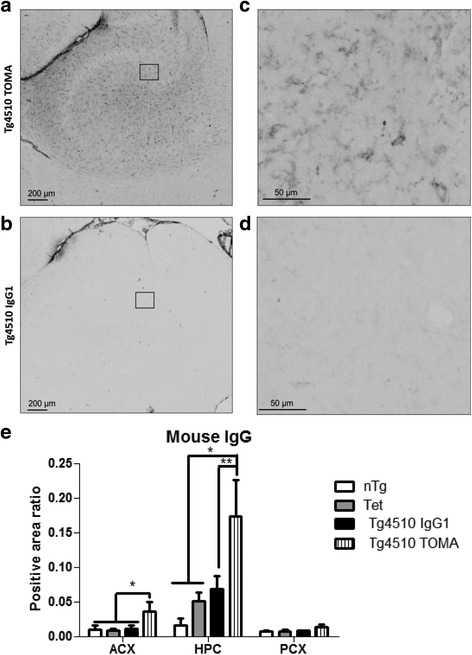
Tg4510 mice injected systemically with TOMA present elevated levels of mouse IgG in the brain. Micrographic representation of mouse IgG staining in hippocampus (*HPC*) of Tg4510 mice treated with TOMA (Tg4510 TOMA, **a**, **c**) or IgG1 (Tg4510 IgG1, **b**, **d**) for 4.5 months. **c**, **d** Magnification of square area in **a** and **b**, respectively. No staining was observed in nontransgenic or Tet-only littermates. Immunostaining quantification (**e**) utilizing Mirax software (Zeiss Inc.) in the anterior cortex (*ACX*), HPC, and posterior cortex (*PCX*) of nontransgenic littermates (*nTg*, *white bars*), tet-only mice (*Tet*, *grey bars*), and Tg4510 mice treated with IgG1 (*Tg4510 IgG1*, *black bars*) or TOMA (*Tg4510 TOMA*, *striped bars*) for 5 months. Two-way ANOVA showed a significant increase in positive area ratio stained for mouse IgG in the ACX and HPC of Tg4510 mice treated with TOMA compared to Tg4510 mice treated with IgG1 and nontransgenic and Tet-only control mice. Data presented as mean ± SEM, *n* = 10/group. *p < 0.05, **p < 0.01. *Scale bar* = 200 μm. *TOMA* tau oligomer monoclonal antibody

Immunohistochemistry was conducted for mouse IgG by incubating sections in anti-mouse IgG specific secondary antibody. Positive peroxidase reaction product staining was observed in the hippocampus and anterior cortex of Tg4510 mice injected with TOMA, but not in Tg4510 mice injected with mouse IgG1 (Fig 1a, b). There was also dark staining of the ependymal regions of the ventricles seen in both treatment conditions. When the parenchymal staining in the hippocampus was viewed at higher magnification, there was staining of the neuropil only in Tg4510 mice injected with TOMA (Fig. 1c, d). We quantified immunostaining in the anterior cortex (ACX), posterior cortex (PCX), and hippocampus (HPC). The ratio of positively stained area to total area calculated from digitized images is shown in Fig. 1e. The Tg4510 mice exhibited significantly higher levels of IgG in the ACX (*p* < 0.05) compared to all other groups, and in the HPC (*p* < 0.05 when compared to IgG1, *p* < 0.01 when compared to the other groups). No significant differences were observed in the PCX.

### Demonstration of TOMA binding to Tau oligomers in Tg4510 mice

**Fig. 2:**
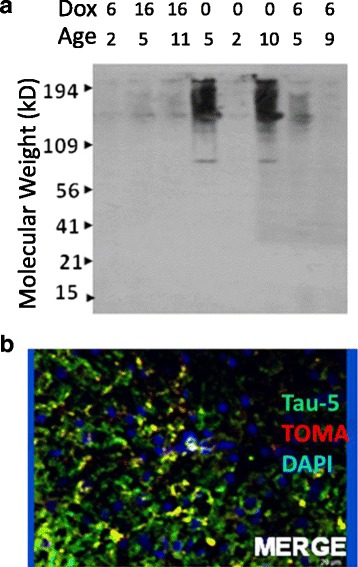
TOMA recognizes high molecular weight tau in material from Tg4510 mice. Western blot image from Tg4510 mouse cortex **a**. Some mice were treated with doxycycline to suppress transgene expression for 6 or 16 weeks (row labeled “*Dox*”). *0*, no doxycycline treatment. Ages of the mice are also indicated in months in the row labeled “*Age*”. Molecular weight marker locations are indicted by *arrows* along the *Y* axis. **b** Merged immunofluorescence micrograph staining for the total tau antibody, Tau-5 (*green*), TOMA (*red*), and DAPI to stain nuclei (*blue*). *Yellow* areas indicate overlap of the Tau-5 and TOMA staining (Color figure online). *TOMA* tau oligomer monoclonal antibody

To confirm that TOMA was capable of binding to the oligomeric forms of tau found in the Tg4510 brain, we first performed western blot analyses using TOMA as primary antibody on three ages of Tg4510 mice with and without treatment with doxycycline to turn off the expression of human tau in these mice. In 5-month-old and 10-month-old Tg4510 mice, high molecular weight oligomers were readily detected by TOMA in untreated mice (Fig. 2a, lanes 4 and 6). At 2 months, before there is tau deposition detected by immunostaining, there was very little high molecular weight material detected (Fig. 2a, lane 5). When doxycycline treatment was applied for 6 or 16 weeks, the high molecular weight TOMA-positive material was reduced at all ages (Fig. 2a, lanes 1–3, 5, and 7–8). This confirms that TOMA does recognize the oligomeric forms of tau in Tg4510 mice and that these oligomeric forms appear to turn over, given their reduction when transgene expression is reduced with doxycycline.

To further demonstrate TOMA binding we performed immunofluorescence using TOMA and Tau-5 as primary antibodies. Tau-5 binds tau at aa210–241 and recognizes all forms of tau. Tau-5 stained numerous profiles in the cortex of Tg4510 mice, as expected (Fig. 2b). TOMA double-labeled a subset of these processes, confirming that it can bind to some of the tau isoforms deposited in the brains of Tg4510 mice at 5 months of age. There was little to no staining with TOMA that was not also labeled by Tau-5. Note also the similar pattern of mouse IgG staining in Fig. 1c from mice injected with TOMA to that when TOMA is used as a primary antibody.

### Detection of oligomeric Tau in plasma and CSF

**Fig. 3:**
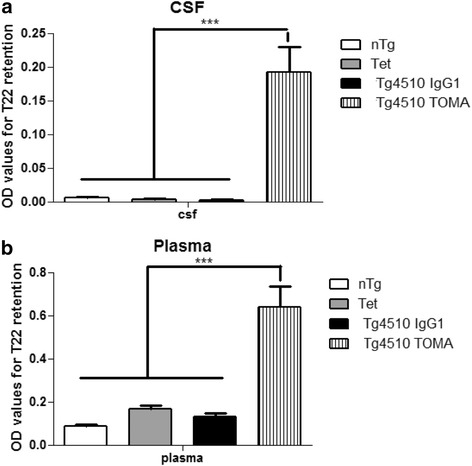
Tg4510 mice injected systemically with TOMA present elevated levels of oligomers in both CSF and plasma. Quantitative analysis of oligomeric tau in *CSF* (**a**) and plasma (**b**) of nontransgenic mice (*nTg*, *white bars*), Tet-only mice (*Tet*, *grey bars*), and Tg4510 mice treated with IgG1 (*Tg4510 IgG1*, *black bars*) or TOMA (*Tg4510 TOMA*, *striped bars*) for 5 months. Analyses were performed using both direct and sandwich ELISA. T22 was used for tau oligomers. Two-way ANOVA showed a significant increase in levels of oligomers in both CSF of Tg4510 mice treated with TOMA compared to Tg4510 mice treated with IgG1 and nontransgenic and Tet-only control mice. Data presented as mean ± SEM, ***p < 0.001. *CSF* cerebrospinal fluid, *TOMA* tau oligomer monoclonal antibody, *OD optical density*

Plasma and CSF were assayed with an ELISA specific for oligomeric tau. In the CSF, oligomeric tau was detected only in the mice injected with TOMA (*p* < 0.001; Fig 3a). In the plasma, the TOMA-treated animals demonstrated significantly elevated levels of oligomeric tau compared to the other three groups (*p* < 0.001; Fig. 3b). These results suggested that TOMA was effectively acting as a sink in both CSF and plasma retaining the oligomeric tau, indicating antibody access to both compartments.

### Behavioral testing

**Fig. 4:**
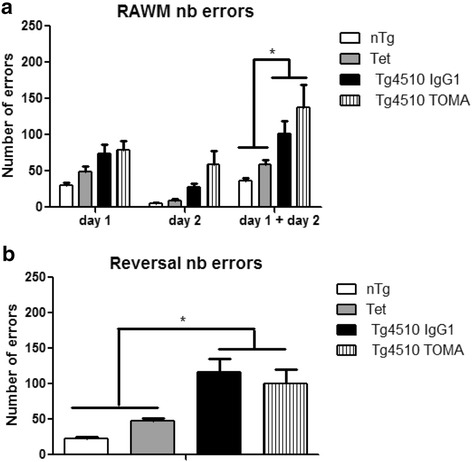
Tg4510 mice presented spatial memory deficits in the RAWM and reversal trial that were not rescued by TOMA treatment. Number of errors during 2-day RAWM (**a**) and reversal (**b**) in nontransgenic mice (*nTg*, *white bars*), Tet-only mice (*Tet*, *grey bars*), and Tg4510 mice treated with IgG1 (*Tg4510 IgG1*, *black bars*) or TOMA (*Tg4510 TOMA*, *striped bars*) for 5 months. Tg4510 mice made significantly more errors attempting to locate a hidden platform in the 2-day RAWM test, compared to nTg mice and Tet mice, regardless of the treatment. Tg4510 mice were not able to learn a new platform location on the reversal trial. Data presented as mean ± SEM, *n* = 10/group. *p = 0.05. *RAWM* radial arm water maze, *TOMA* tau oligomer monoclonal antibody, nb Number

There were no overall ANOVA differences in the open field, rotarod, or Y-maze tasks for any of the comparisons (data not shown). Mice were tested for spatial navigation performance in the radial arm water maze. This is a variant of the Morris water maze with a submerged platform at the end of one of six swim alleys, permitting errors to be measured rather than time. Over the first 2 days of training, there was a significant ANOVA effect with both groups of Tg4510 mice requiring more errors to find the platform than nTg or Tet mice (Fig. 4a). On the third day of testing, the platform was placed in the arm opposite to that used for learning on the first 2 days. Again, the ANOVA was significant with more errors on this reversal task for both groups of Tg4510 mice than for the nTg or Tet mice (Fig. 4b). In neither the learning nor reversal portions of the training were the TOMA-treated Tg4510 mice different from the IgG1-treated mice.

**Fig. 5:**
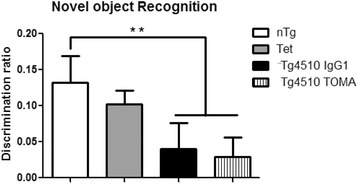
Tg4510 mice presented short memory deficits during the novel object recognition test that were not rescued by TOMA treatment. Percentage of exploration (calculated as the time spent with the novel object divided by the sum of times spent with novel and familiar, multiplied by 100) of the novel object presented in the last trial of the novel object recognition test in nontransgenic mice (*nTtg*, *white bars*), Tet-only mice (*Tet*, *grey bars*), and Tg4510 mice treated with IgG1 (*Tg4510 IgG1*, *black bars*) or TOMA (*Tg4510 TOMA*, *striped bars*) for 5 months. Tg4510 mice spent significantly less time exploring the novel object compared to nontransgenic littermates, regardless of the treatment. Data presented as mean ± SEM, *n* = 10/group. **p < 0.01. *TOMA* tau oligomer monoclonal antibody

Mice were also examined for novel object recognition memory. In this task, mice exposed to a novel object and a familiar object are expected to spend more time attending to the novel object. Overall ANOVA and means testing determined that the Tg4510 mice spent significantly less time attending to the novel object than did nTg or Tet mice (Fig. 5). Again there was no significant difference between the TOMA-treated and IgG1-treated Tg4510 mice.

### Histology

**Fig. 6:**
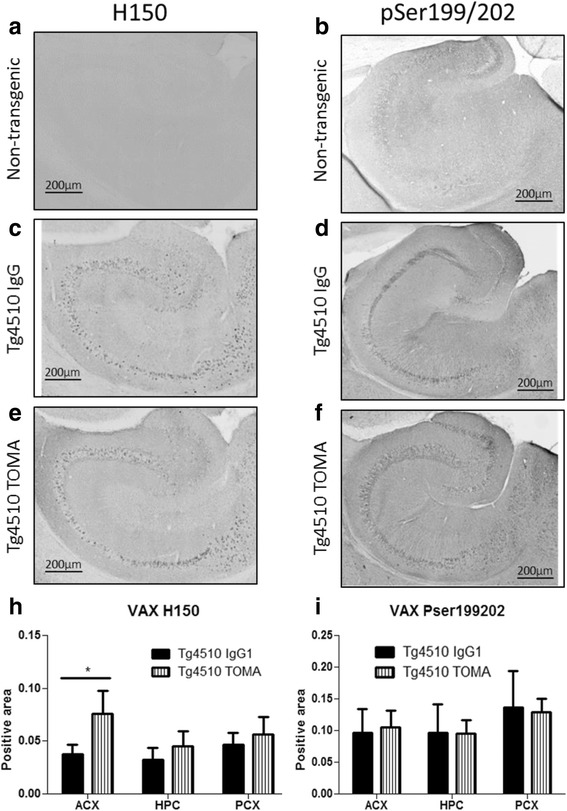
Treatment with TOMA did not affect levels of tau detected by antibody pSer396 or Gallyas silver staining. Representative sections from nontransgenic mice (**a**, **b**), Tg4510 mice treated with IgG (**c**, **d**), or Tg4510 mice treated with TOMA (**e**, **f**) for the hippocampus. Little staining is detected in the nontransgenic mice (**a**, **b**) or Tet mice (data not shown). Immunostaining quantification utilizing Mirax software (Zeiss Inc.) in the anterior cortex (*ACX*), hippocampus (*HPC*), and posterior cortex (*PCX*) of Tg4510 mice treated with IgG1 (*black bars*) or TOMA (*striped bars*) (**h**, **i**). *p < 0.05. Data presented as mean ± SEM. *TOMA* tau oligomer monoclonal antibody

**Fig. 7:**
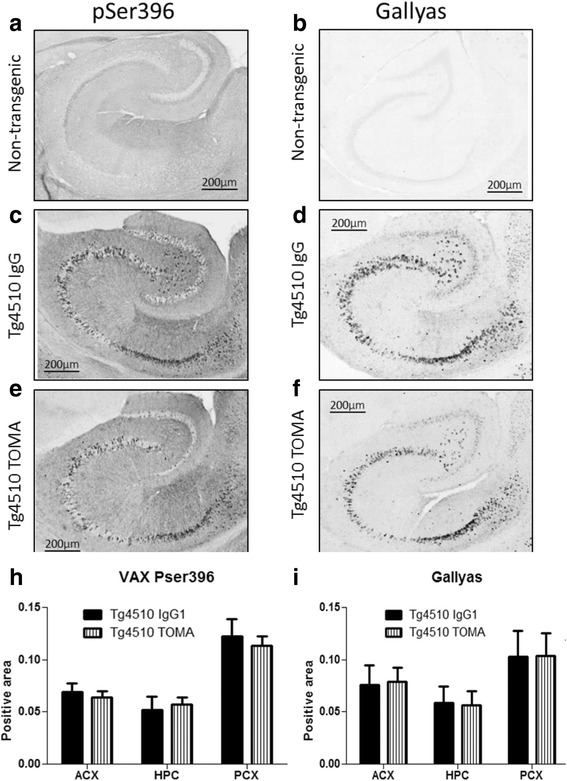
Treatment with TOMA did not affect levels of tau detected by antibody H150 or pSER199/202. Representative sections from nontransgenic mice (**a**, **b**), Tg4510 mice treated with IgG (**c**, **d**), or Tg4510 mice treated with TOMA (**e**, **f**) for the hippocampus. Little staining is detected in the nontransgenic mice (**a**, **b**) or Tet mice (data not shown). Immunostaining quantification utilizing Mirax software (Zeiss Inc.) in the anterior cortex (*ACX*), hippocampus (*HPC*), and posterior cortex (*PCX*) of Tg4510 mice treated with IgG1 (*black bars*) or TOMA (*striped bars*) (**h**, **i**). Data presented as mean ± SEM. *TOMA* tau oligomer monoclonal antibody

One half of the brain was sectioned for histochemical analysis. We used several different approaches to detecting tau deposits in these mice. The antibody H150 is directed against the N terminus of tau and detects all forms of tau. A second antibody is directed against the phosphorylated tau epitope at positions 199/202 (overlapping the AT8 epitope, an early marker of tau transformation). In these 8-month-old mice, both markers readily labeled neurons in the hippocampus of both TOMA-treated and mouse IgG-treated Tg4510 mice, with minimal staining of nTg animals (Fig. 6). When quantified by image analysis there were no significant differences in immunopositive area between TOMA and control treated mice in the anterior cortex, hippocampus, or posterior cortex, except for increased H150 staining in the anterior cortex of TOMA-treated mice (Fig. 6h, i). This increase appeared due to two TOMA-treated mice with unusually heavy staining restricted to this region. Additionally we stained tissue sections for tau phosphorylation at residue 396, overlapping the PHF-1 epitope, a late stage marker. We also stained for neurofibrillary tangles using the Gallyas silver stain method. Tg4510 mice at 8 months of age have numerous pSer396 and Gallyas-positive neurons throughout the hippocampus in both pyramidal and granule cell layers (Fig. 7c–f). No Gallyas-positive cells were found in tissues from nTg (Fig. 7a, b) or Tet mice (data not shown). When the amount of staining was quantified in the anterior cortex, hippocampus, and posterior cortex, there were small regional differences but no effect of TOMA treatment on the development of this pathology (Fig. 7h, i).

**Fig. 8:**
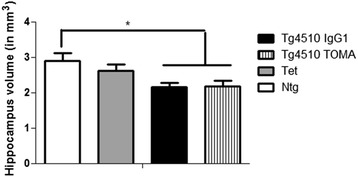
Hippocampal atrophy in Tg4510 mice is unaffected by treatment with TOMA. Hippocampal volume was estimated from tissue sections through the entire hippocampus using the method of Cavalieri. Volume is expressed in cubic millimeters. Tg4510 mice treated with TOMA or IgG are significantly reduced relative to nontransgenic mice (*p < 0.05). Tet mice are not different from any of the other groups. Data presented as mean ± SEM. *Ntg* nontransgenic mice, *TOMA* tau oligomer monoclonal antibody, *Tet* Tet-only mice

One impact of the tau pathology in Tg4510 mice is loss of hippocampal volume. We measured the hippocampal volume using the method of Cavalieri [26]. As we found in prior studies, 8-month-old Tg4510 mice have reductions in hippocampal volume of 25% compared to nontransgenic mice (Fig. 8). However, there was no effect of antibody treatment on this volume loss.

We further analyzed all results separately for male and female mice. We did not detect any effects of TOMA treatment in either the male or female groups, and thus data are presented in aggregate. The only marker for which the female mice had more pathology was Gallyas staining, in which the female mice had roughly twice the male mouse values.

### Western blotting

**Fig. 9:**
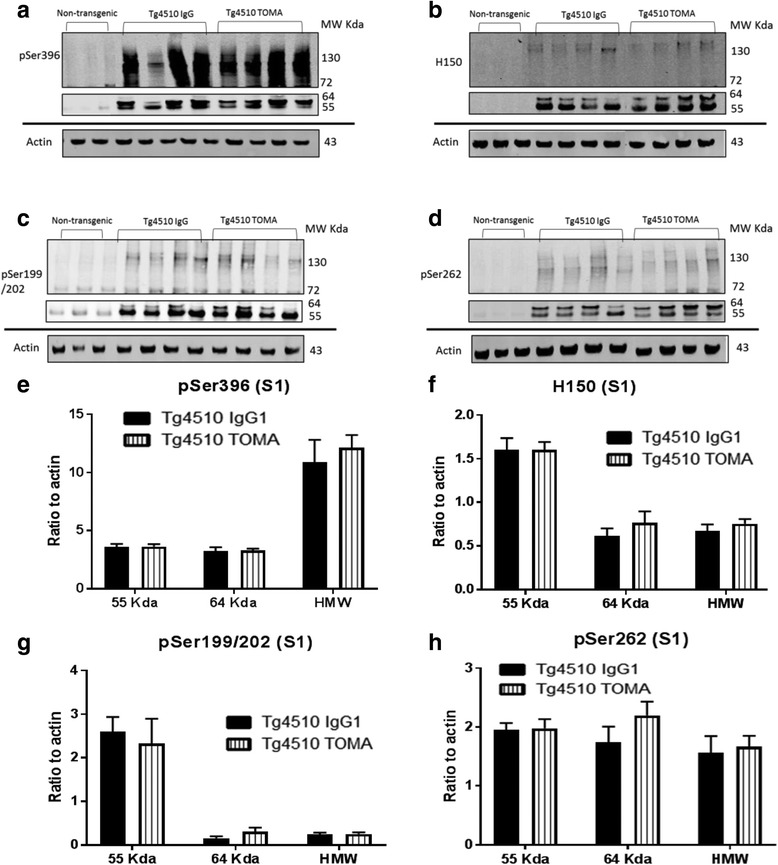
Treatment with TOMA did not affect soluble tau detected by western immunoblotting analysis. Anterior cortex was homogenized in RIPA buffer and the detergent soluble fraction prepared by centrifugation of the homogenate. **a**–**d** Representative immunoblots for antibodies pSer396, H150, pSer 199/202, and pSer262 respectively. Data were quantified for three molecular weight size ranges for each antibody and normalized to actin (**e**–**h**). Data expressed as mean ± SEM. *TOMA* tau oligomer monoclonal antibody.* MW Kda *molecular weight in kilodaltons. *HMW* high molecular weight (100-160 Kda)

The detergent soluble fraction (S1) of homogenized tissue from the right ACX was analyzed by western blot analysis. We used the same antibodies as used for immunostaining (H150; pSer199/202 and pSer396) and added an antibody directed against tau phosphorylated at residue Ser262, within the microtubule binding domain and believed to interfere with microtubule binding. We separately quantified the bands at 55, 64, and 120–200 kDa (likely representing oligomers) and normalized these values to actin (Fig. 9). We found no differences between the IgG1 control and the TOMA treatment using these detection antibodies (Fig. 9e–h).

**Fig. 10:**
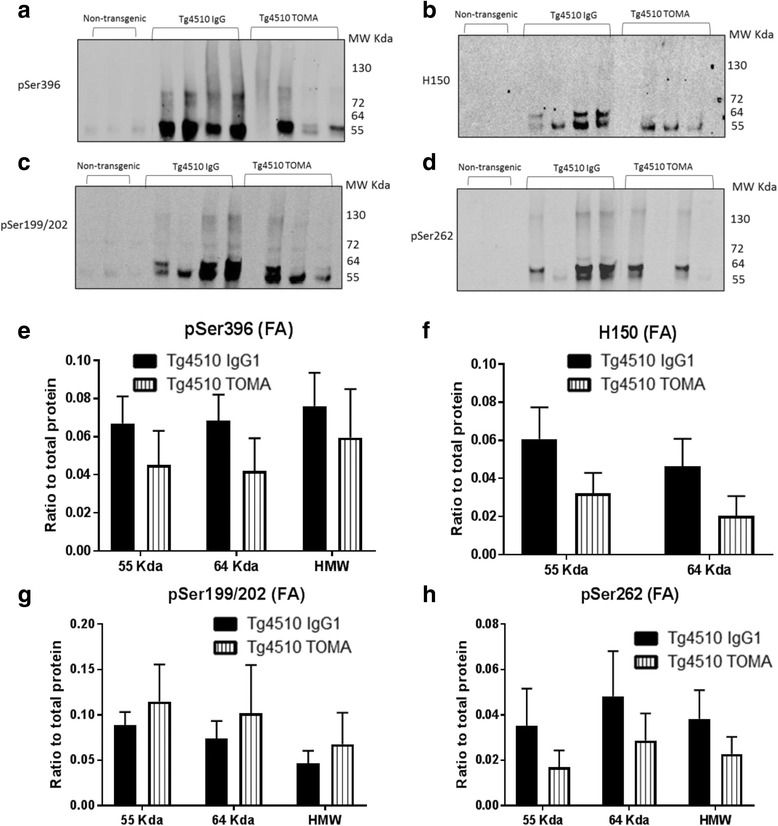
Treatment with TOMA did not affect insoluble tau detected by western immunoblotting analysis. Anterior cortex was homogenized in RIPA buffer and the detergent insoluble fraction prepared by centrifugation of the homogenate. The pellet was solubilized with formic acid and neutralized with NaOH prior to electrophoresis. **a**–**d** Representative immunoblots for antibodies pSer396, H150, pSer 199/202, and pSer262 respectively. Data were quantified for three molecular weight size ranges for each antibody and normalized to total protein visualized with the Revert reagent (**e**–**h**). Data expressed as mean ± SEM. *TOMA* tau oligomer monoclonal antibody. *MW Kda* molecular weight in kilodaltons. *HMW﻿ *high molecular weight (100-160 kilodaltons)

The detergent insoluble tissue fraction was digested with formic acid and then neutralized using NaOH, to permit analysis of tau components as insoluble tangles or other detergent insoluble intermediates. Unlike the soluble fraction, individual mice have considerable variability in the amounts of insoluble tau detected, consistent with prior observations (normalized by total protein using the Revert reagent (LiCor), because actin or GAPDH are not detected in this fraction). There were no significant differences between the IgG-treated and TOMA-treated mice with any antibody at any molecular weight band (Fig. 10).

## Discussion

Numerous studies have been conducted, to date, utilizing various immunotherapy strategies in an attempt to prevent the development of a tau phenotype in a variety of preclinical models. We have recently reviewed this literature and included some of our own data in investigating intracranial delivery of immunotherapy agents [9]. Here, we focused on an oligomeric tau-targeted antibody as passive immunotherapy for Tg4510 mice. We evaluated the tau phenotype in these mice with respect to behavior, histology, and biochemical markers of pathology in attempt to identify whether TOMA would be a potential therapeutic candidate [27]. On the whole, our studies showed that in this mouse model TOMA was not as effective as has been demonstrated in previous studies using different models of tauopathy [17, 28, 29].

One possible explanation for an absence of impact on the tau phenotype could be failure of the antibody delivery or altered pharmacokinetics. We were able to observe elevated Tau oligomers both in CSF and in plasma of mice treated with TOMA, but not in mice treated with mouse IgG1. The most probable explanation for these elevations is the binding of tau oligomers to the antibody in these compartments and the delay in its clearance due to being bound by antibody. Such a mechanism has been proposed to explain elevated circulating Aβ levels after treatment with some antibodies [30–32]. Other antibodies targeting monomeric tau have also been reported to elevate plasma tau [33]. However, a different study found antibodies specific for phospho-tau treated for 12 weeks decreased tau levels in CSF (associated with declines in tissue tau levels [34]).

A second explanation may be the poor penetration of antibody into brain parenchyma. However, we detected mouse IgG decorating the neuropil of hippocampus and cerebral cortex, sites of tau expression in Tg4510 mice. This staining in general did not appear to be within neurons, but associated with the molecular layers in hippocampus. It is conceivable that the absence of an effect was due to the inability of this antibody to become internalized by neurons, as appears to be the case for some anti-tau antibodies [35, 36]. However, given the specificity of the antibody for oligomeric tau, it may represent the locations of this antigen extracellularly or on the cell surface. Again we did not observe this staining in mice injected with IgG1 control antibodies. These results suggest that at least some antibody reached the brains of the Tg4510 mice.

The results from behavioral tests indicated the expected genotype effect of the Tg4510 mice as having worse performance than the nTg or the Tet control mice. We included the Tet group because of prior work showing that the Tet transgene alone can result in a phenotype, including slightly reduced hippocampal volume [37]. When we compared the IgG1-treated and TOMA-treated Tg4510 mice, there was no significant difference, indicating that TOMA was not able to prevent the cognitive deficits in the radial arm water maze, either in the initial learning phase or in the reversal phase. These results were unexpected based on the behavioral improvements in Y-maze performance in the initial publication of this treatment [17]. However, those studies used the JNPL3 mouse model, not the Tg4510 mice. The novel object recognition (NOR) results were consistent with the radial arm water maze results, indicating a genotype effect but no treatment effect. These results contrast with those reported by Castillo-Carranza et al. [28], who did identify that immunotherapy with TOMA led to an improvement in NOR using htau mice seeded with preformed tau aggregates to induce pathology and behavioral deficits.

The results from immunohistochemistry or Gallyas staining do not show any significant differences between the treatments for the three regions inspected, except for one region with one antibody, perhaps not unexpected given the number of comparisons made in this study. In any event, the direction of this change did not favor the hypothesis that TOMA should reduce pathology. Overall, these data suggest that there was little to no effect of TOMA on parenchymal levels of different forms of tau or Gallyas positive neurofibrillary tangles. Other work with the TOMA antibody has failed to detect reductions in AT8 staining or Gallyas staining in JNPL3 (P301L) tau transgenic mice [17], or in htau mice injected with tau seeds [28]. It was argued that the behavioral deficits were due to tau-oligomers and their removal was sufficient to rescue behavioral deficits.

We examined four markers of tau pathology via western blot analysis to quantify changes in different tau fractions and molecular weight variants. We examined both detergent soluble tau and detergent insoluble tau (solubilized with formic acid) fractions. We were unable to detect treatment effects in the two tau monomer bands we could discern (55 and 64 kDa), nor in the higher molecular weight forms of tau believed to represent oligomers. This latter observation is in contrast to prior work in the JNPL3 and htau models [17, 28].

There were several methodological differences between the present results and those demonstrating successful rescue of cognitive changes with TOMA. One is the route of antibody administration. Prior work performed intravenous administration via the tail vein. All of our prior efforts using passive systemic immunotherapy in mouse models of Alzheimer’s pathology (largely anti-Aβ studies) successfully administered antibodies via intraperitoneal injections [32, 38–40]. Other potential explanations for the discrepancies could be that the Tg4510 mouse is sufficiently aggressive that higher antibody doses would be required to effectively lower the tau oligomers to rescue the cognitive impairments. The Tg4510 mouse deposits tau earlier than most other tau transgenic mice and has greater neurodegeneration than most other models [41]. However, a third possibility is that the cause of the cognitive impairments in the Tg4510 mouse is different than in JNPL3 or htau models. By 6 months, there is considerable loss of hippocampal neurons and cortical atrophy in Tg4510 mice [19, 42]. The data obtained previously with TOMA result in relatively rapid reversal of the cognitive impairment, implying that the rescue is not due to protection from structural changes, but impairments of synaptic function that are reversible. However, it is feasible that the Tg4510 mouse has cognitive deficits which are secondary to neurodegeneration and brain atrophy. Removing tau oligomers may not improve cognition because sufficient neural substrates for cognition no longer exist at 8 months. The atrophy would not be expected to be reversed rapidly, but may require protracted administration from before the age when pathology begins. Thus, treating from 3.5 to 8 months may not be adequate to provide this protection. It is conceivable that had we tested the mice at an earlier age (e.g., 5 months) the cognitive deficits might have been more susceptible to clearance of oligomeric tau.

Our overall experience has been that preventing pathology in the Tg4510 mouse is challenging. Some of our negative results have been published [20, 23, 43] while others have not. The few instances in which we obtained positive effects of treatment have required direct and long-term application to the brain of genes expressed at high levels resulting in reduced tau deposition [42, 44]. To our knowledge, the only study demonstrating a significant impact of passive immunotherapy in the Tg4510 model was that by Sankaranarayanan et al. [34]. These authors treated Tg4510 mice weekly (as opposed to biweekly here) with 25 mg/kg (10 times more than used here) of two phospho-tau specific antibodies from 3 to 6 months. They detected a 20% decline in soluble AT8. However, there was no decline in detergent insoluble total tau or AT8 tau isoforms. In parallel studies, they examined the PS19 (P301S) tau mouse and observed much larger reductions in tau pathology, on a percentage basis.

## Conclusions

Tau immunotherapy remains an attractive area of investigation for the treatment of AD and other tauopathies. However, the wide range of preclinical tauopathy models, each having its own characteristics, and also the wide range of immunogens administered to date confound the field. The goal of this avenue of research is to ultimately identify a treatment for tauopathies such as AD, but that bullseye treatment has not yet been identified. Synergy between tau and Aβ treatments would likely provide a most useful avenue for further investigation into AD treatments, because recently TOMA was found to benefit an APP mouse model [29] (but see also [45]). Although the Tg4510 model rigorously replicates the extensive neuron loss and atrophy of AD, it may be prudent to reserve this model only for treatments already demonstrated effective in other preclinical tauopathy models and to initiate treatment at very early stages.

## Abbreviations

ACX: Anterior cortex
AD: Alzheimer’s disease
CSF: Cerebrospinal fluid
HPC: Hippocampus
nTg: Nontransgenic
PCX: Posterior cortex
Tet: Tetracycline response element transgenic
TOMA: Tau oligomer monoclonal antibody
